# HL7 FHIR with SNOMED-CT to Achieve Semantic and Structural Interoperability in Personal Health Data: A Proof-of-Concept Study

**DOI:** 10.3390/s22103756

**Published:** 2022-05-15

**Authors:** Ayan Chatterjee, Nibedita Pahari, Andreas Prinz

**Affiliations:** 1 Department of Information and Communication Technology, Center for eHealth, University of Agder, 4630 Kristiansand, Norway; andreas.prinz@uia.no; 2 Department of Software Development, Knowit As, 4836 Arendal, Norway; nibedita.pahari@knowit.no

**Keywords:** HL7, FHIR, SNOMED-CT, PHR, PGHD, PHR-S FM, interoperability, eCoach, TSD

## Abstract

Heterogeneity is a problem in storing and exchanging data in a digital health information system (HIS) following semantic and structural integrity. The existing literature shows different methods to overcome this problem. Fast healthcare interoperable resources (FHIR) as a structural standard may explain other information models, (e.g., personal, physiological, and behavioral data from heterogeneous sources, such as activity sensors, questionnaires, and interviews) with semantic vocabularies, (e.g., Systematized Nomenclature of Medicine—Clinical Terms (SNOMED-CT)) to connect personal health data to an electronic health record (EHR). We design and develop an intuitive health coaching (eCoach) smartphone application to prove the concept. We combine HL7 FHIR and SNOMED-CT vocabularies to exchange personal health data in JavaScript object notion (JSON). This study explores and analyzes our attempt to design and implement a structurally and logically compatible tethered personal health record (PHR) that allows bidirectional communication with an EHR. Our eCoach prototype implements most PHR-S FM functions as an interoperability quality standard. Its end-to-end (E2E) data are protected with a TSD (Services for Sensitive Data) security mechanism. We achieve 0% data loss and 0% unreliable performances during data transfer between PHR and EHR. Furthermore, this experimental study shows the effectiveness of FHIR modular resources toward flexible management of data components in the PHR (eCoach) prototype.

## 1. Introduction

### 1.1. Overview

As defined by the National Health IT Coordinator, personal and/or person-generated health data (PGHD) are created, and recorded by individuals, family members, or caregivers either passively or continuously to create an accurate and comprehensive picture of health and health plans, (e.g., decision support, recommendation generation, diagnostics, treatment plan) [1,2,3]. Individuals cannot directly access their health data stored inside EHR. The primary stakeholders are participants or patients interested in acquiring their data to monitor chronic illness or track fitness levels and engage more in their health wellness evaluation. They consent to process or share their personal health information with actual providers. PGHD is maintained and processed by primary healthcare professionals and researchers on the provider side. PGHD includes health history, biometric data, symptoms, medication effects, physiological data, behavioral data, (e.g., sleep, diet, habit, work, and exercise), lifestyle choices, patient-reported outcome measures, and treatment history. The sources of PGHD can be mobile applications, wearable devices, registered medical grade devices, interviews, surveys, questionnaires, or any other interactions with technology that produce personal data about health. PGHD is the key to improving health outcomes, care delivery, and patients’ lives, and therefore should be part of any evidence generation strategy. The U.S. Food and Drug Administration (FDA) recognizes the vital role of these data, identifies PGHD as one of the primary sources of real-world data, and recommends creating real-world evidence for critical analyses of randomized trials and observational studies using PGHD. PGHD has been essential to provide deeper insights for developing new medical products and providing more human-centered care [1,2,3,4,5,6,7,8].

According to the Markel Foundation, the PHR is, “an electronic application through which individuals can access, manage, and share their health information, and that of others for whom they are authorized, in a private, secure, and confidential environment” [9]. Electronic medical record (EMR) is generally regarded as an internal organizational system, while EHR is defined as a cross-organizational system [9]. An EMR–tethered PHR system can engage patients more actively in managing their chronic conditions with lifelog data [10]. HL7 PHR-S FM [11] is a set of features and functions needed for PHR. HL7 PHR-S FM is neither a PHR data model nor an implementation but a framework that lists the required or desired tasks in the PHR. Experts can use PHR-S FM as a frame of reference to design and implement PHR. The PHR types can be divided into the following three categories [11]: individual (a separate or standalone application that acts as a digital journal, such as MyMedical, Microsoft HealthVault that collects personal health data; however, does not communicate with EHR), tethered (a PHR is connected to a single EHR and the EHR data is harmonized with PHR based on the consented permission), and integrated (a PHR is synchronized with multiple sources, such as home diagnostics, EHRs, pharmacy, and insurance systems). According to the experts and researchers, tethered PHR provides greater effectiveness than individual PHR, and the evidence has been collected from the unsuccessful Google Health project [11].

### 1.2. Motivation

Semantic and structural interoperability is designed to share electronic health data across all departments of an organization, clinicians, nurses, laboratories, and the entire hospital. A semantically and structurally integrated healthcare system enables data to be communicated between organizations and their internal ecosystems without losing the meaning of domain concepts, contextual knowledge, and formal data representations. Therefore, semantic, and structural interoperability avoid data silos and keep data providers independent for exchanging information across organizational boundaries. However, finding semantic and structural interoperability in health records and disparate clinical annotations is one of the biggest challenges and a constant research goal in recent years. Structural and semantic integration is the ability of two or more systems or components, (e.g., PHR, EHRs) to exchange information and consistently represent them in a uniform way [12]. A semantic integration between a PHR and EHRs allows stakeholders to describe requirements regardless of technical implementation [12]. A semantic and structural integration following a guideline in the bi-directional communication between EHR and PHR has brought the attention of interdisciplinary researchers. Hussein et al. [13] emphasized the vital role of interoperability in developing and adopting a fully functional PGHD-enhanced health ecosystem to exchange unified data among patients or participants, healthcare providers, and researchers. Through its DH-Convener project, they supported PGHD interoperability and security with an eye toward the associated clinical-technical user aspects [13]. Plastiras et al. [14] developed a middleware framework for facilitating PHR and EHR interoperability based on ontologies. Rohrs et al. [15] proposed an application model to ease integration issues caused by the lack of interoperability between different standards to provide a single updated point-of-view PHR. According to Urbauer et al. [16], interoperability specifications of standard-based communication of systems and personal health devices have contradictions and gaps that could be resolved. Likewise, organizations proposed different standards to make interoperability easier, and some prominent means have been translated into unified modeling language (UML) to facilitate model-driven healthcare application development [17,18,19]. As a result of SMART on FHIR specification, Sayeed et al. [20] created SMART Markers, a mobile device software framework that can be used to deploy both patient-facing and practitioner-facing apps for PGHD. Their approach creates context-specific experiences that support the integration of health systems for both patients and practitioners. HL7 FHIR serves as the single data model in FHIR as an application programming interface (API) [9].

HL7 FHIR offers a modular approach to healthcare data representation instead of the traditionally document-centric approach as autonomous substances called resources [9]. The HL7 FHIR standard is planned to utilize existing HL7 standard/model(s) and major innovative redesign using the current web and versatile advancements, such as the lightweight HTTP-based REST (representational state transfer) convention, JavaScript object notion (JSON), extensible markup language (XML), and resource description framework (RDF) [9]. The HL7 Version 2 (V2) Messaging standard 7 supports granular information payloads and benefits many organizations [21]. FHIR is neither a security convention nor characterizes any security-related functionalities [22]. Therefore, data exchange through HL7 FHIR API must be protected from external vulnerabilities.

### 1.3. Aim of the Study

A proof-of-concept (PoC) implements a method or idea to demonstrate its feasibility or a proof-of-principle to test whether a concept or theory has actual potential. The scope of a PoC can be limited; however, it focuses on the targeted population. This study has focused on integrating an information model and clinical terms to establish a structural and semantic consistency between the PHR (the eCoach mobile app) and the EHR (a database inside TSD) following a standard guideline, (e.g., PHR-S FM). Here, PGHDs have been collected with the PHR (an eCoach mobile app) and recorded into the EHR (a TSD database or PostgreSQL). HAPI FHIR server has been operative between the PHR and the EHR to provide a structural and semantic consistency of bi-directional communication. TSD’s authentication and authorization method have secured the entire communication channel. This study addresses the following research questions:

***(RQ-1)** How to connect personal health data to an EHR using structural and semantic interpretation?*

***(RQ-2)** How to verify that the tethered PHR implementation has followed PHR-S FM functional standards?*

***(RQ-3)** How to verify that there are no data loss and performance drops during the data transfer between PHR and EHR across different devices, such as smartphones, and desktop web?*

We have used HL7 FHIR, SNOMED-CT, PHR-S FM, and TSD (as Infrastructure as a Service or IaaS) to design and develop a tethered eCoach PHR application to address the research questions. TSD helps to achieve Microservice API (application programming interface) security during the external data exchange. 

## 2. Related Work

This section presents existing knowledge applicable to current research. We performed a literature search with the following search string pattern: (personal health data OR patient health data OR patient-generated health data OR person-generated health data) AND (personal health record OR patient health record OR PHR) AND (electronic health record or EHR) AND (HL7 OR FHIR OR HAPI FHIR OR CCR OR CCD OR CDA) AND (SNOMED-CT or SNOMED CT OR LOINC) AND (Integration OR Interoperability) on the following electronic databases—Scopus, EBSCOhost, IEEE Xplore, Nature, Science Direct, PubMed, IEEE, Google Scholar, and SpringerLink. A subset of these articles is cross-referenced between portals, especially Google Scholar and PubMed. Related search keywords were identified using terms of MeSH (Medical Subject Headings), synonyms, relevant articles, and self-determined search terms. We used EndNote (V. X9), DOAJ, Sherpa/Romeo, and Microsoft Excel (MS Office 365 V. 16.x) to efficiently search, collect, and select related articles. We included articles based on the following inclusion criteria: (1) peer-reviewed, full-length articles written in English, and (2) articles published in the selected databases between 2014 and 2021. We excluded studies related to EHR interoperability, core ontology-based semantic searching or search engines, and artificial intelligence-based text processing for the latent semantic analysis [23,24,25]. Therefore, the search resulted in peer-reviewed, full-length articles on PHR architectures, models, and systems/implementations with or without semantic standards.

### 2.1. Structural Standards

Hommeaux et al. [26] developed a Java-based FHIR resource description framework (RDF) for PHR data transformation and validation. RDF is a standardized HL7 FHIR data format. Furthermore, they created a verification framework based on RDF Shape Expressions (ShEx) to verify FHIR RDF data. The framework conformed to the ShEx model provided in the FHIR specification of FHIR versions R4 and R5. Gulden et al. [27] investigated how the HL7 FHIR can be used as a standard format for exchanging and storing clinical trial records. The results showed that FHIR resources established a unified view of research information from heterogeneous sources by realizing automatic data exchange between the test center and the central research registry. Mandl et al. [28] described the specification of the SMART/HL7 FHIR bulk data access API, which established access to patient-level data for the entire patient population and supported countless use cases across healthcare, research, and public health ecosystems. The API supported “button-type population health” as the core data elements can be quickly and standardly extracted from electronic health records, enabling local, regional, and national data-driven innovation. Kiourtis et al. [29] proposed a mechanism to aggregate the semantic and syntactic similarity in healthcare data and transform it into corresponding HL7 FHIR architecture to promise healthcare interoperability. They further verified the quality of the proposed mechanism with aligned APIs and a non-dominated sorting genetic algorithm (NSGA-III) to achieve ontology alignment. Hussain et al. [30] described the advantage of HL7 protocols for annotating SIIM image Hackathon Dataset as a PoC study using the HAPI FHIR server to achieve interoperability. They demonstrated the FHIR server compilation and installation prerequisites in the Apache Tomcat web server and SIIM medical image data loading through HAPI REST API. Plastiras et al. [31] developed a standardized information model based on HL7 standards for interoperability to support PGHD and observations of daily living (ODL) data types. The extended data models utilized HL7 Clinical Document Architecture (CDA) to address information and instantiated as a Protégé ontology to exchange information among PGHD systems to encourage patient self-management. Kyazze et al. [32] developed a ubiquitous PHR on top of Microsoft HealthVault, accessible via mobile or web, to enable first-time patients to communicate their healthcare data recorded using the PHR to the providers. Bloomfield et al. [33] and Mandel et al. [21] implemented Substitutable Medical Apps and Reusable Technologies (SMART) on HL7 FHIR compatible server infrastructure to enable a successful integration between consistent providers and patient-facing medical apps. 

### 2.2. Open-Source Solutions

Gruendner et al. [34] developed a preprocessor to use standard open-source tools, including PostgreSQL (PSQL), to store FHIR data in a format that can generate further filtering and grouping, and feature selection mechanisms. Verma et al. [35] summarized the impact, opportunities, and challenges associated with the Open-source record Medical Record System (OpenMRS) as a global good. OpenMRS has been implemented in 62 countries worldwide since 2004 for quality healthcare delivery, improved healthcare processes, better reporting and decision making, improved data availability, and enhanced interoperability. Zong et al. [36] proposed an FHIR-based framework based on a case study with colorectal cancer data to model clinical case report form (CRF) data for clinical trials and downstream applications. Pfaff et al. [37] developed an open-source Clinical Asset Mapping Program (CAMP) for FHIR (CAMP FHIR) to efficiently transform and store clinical data into Common Data Model (CDM) to support source-agnostic CDM-to-FHIR-mapping. Saripalle et al. [11] explored HL7 FHIR to design and prototype an interoperable mobile PHR that conforms to the HL7 PHR functional model and allows two-way communication with OpenEMR. Li et al. [38] used HL7 CDA, an XML-based document standard with a steep learning curve and complex architecture, and designing an HL7 CDA configuration file is not a simple process. Compared with FHIR, there is little support for HL7 CDA and its message format. Any available tools/libraries (such as OpenHealthTools MHDT, Everest Framework, etc.), may not be well documented and maintained. Rohers et al. [39] developed OmniPHR, a distributed fastened PHR utilizing openEHR, distributed ledger technology, and other administration ideas that permit patients to keep up with their wellbeing history in a holistic view from any gadgets in a ubiquitous manner. Cerón et al. [40] designed and implemented a PHR system using the Indivo model to monitor blood glucose monitoring in diabetes mellitus type 2 patients. The Indivo architecture is document-centric, with a document model adapted for information needed by patient-centric applications. The document data model is open and available as part of the Indivo open-source suite. The application can also handle information on Continuity of Care Record (CCR) and Continuity of Care Document (CCD).

### 2.3. Security Solutions

Lee et al. [41] developed a blockchain-based architecture to ensure PHR security, integrity, confidentiality, availability, and secure international, cross-institutional, and internal exchange of health records using HL7 FHIR international standards as data format. Margheri et al. [42] designed and implemented a blockchain-based secure decentralized platform for tracking and exchanging patients’ health records following the patient-informed consent preferences and the latest healthcare standards. To achieve interoperability, they integrated the standards, such as HL7, FHIR, and Integrating the Healthcare Enterprise (IHE). They combined their decentralized platform with the SpiritEHR engine of the enterprise-level healthcare system and stored and retrieved available documents in the CDA repository. Hylock et al. [43] formulated a patient-centered mixed blockchain framework to increase patient engagement, data curation, data privacy and confidentiality, and secure data exchange with HL7 FHIR resources. 

### 2.4. Semantic Standards and Data Exchange

Odigie et al. [44] developed a clinical system to represent imaging-related clinical evidence logic statements (CELTS) using semantic standards, such as SNOMED CT, FHIR, and clinical quality language (CQL) to adopt the promotion and acceleration of evidence-based practice. Zhang et al. [45] performed a semantic integration of clinical laboratory tests from PHRs using LOINC codes for deep phenotyping and biomarker discovery. They transmitted the encoded data in FHIR standards after mapping the LOINC encoded data into Human Phenotype Ontology (HPO) terms. Hawig et al. [46] designed a distributed ledger technology (DLT) system with public IOTA (a directed acyclic graph-based DLT) and a private interplanetary file system (IPFS) cluster for the exchange of glucose data following the FHIR semantic standards and GDPR. Tao et al. [47] introduced a bespoke system—Epilepsy Tracking and optimized Management engine (EpiToMe) using HL7 (v2.3) messaging exchange engine to ease the burden of care management in epilepsy by optimizing patient care documentation and clinical workflow.

### 2.5. Novel Contribution and Qualitative Comparison

Security and data privacy are crucial in exchanging data between different healthcare applications; however, they are missing in many studies. Most PHRs use complex document-centric standards, such as CCD, CDA, and ASTM CCR, to exchange data among heterogeneous healthcare systems and applications to promise interoperability. However, HL7 FHIR is new, flexible, easy to use, and based on the web-based technology interoperability standard. This study focuses on designing and implementing a secure tethered PHR to connect personal health data to an EHR, following the norms and functional requirements. Further studies related to PHR have focused on the following PHR types: standalone, tethered, and integrated (see Table 1). 

To implement a tethered PHR following the PHR-S FM functional requirement, we have addressed interoperability with HL7 FHIR and SNOMED-CT vocabularies. Moreover, we have used TSD-based authentication and authorization mechanism to secure the PHR. As a standard in PHR design and development, PHR-S FM functional requirement is missing in the existing literature except in the study conducted by Saripalle et al. (see Table 1). However, our analysis incorporates some extended functionalities as specified in the Method section. Different studies are performed on various platforms, and they did not address the implementation on a detailed level. Thus, a quantified comparison between our research and the existing research has been challenging. However, a qualitative comparison between our work and the relevant previous works is shown in Table 1 and discussed in the Discussion Section. In qualitative comparison, the key parameters are integration standards, security and authentication, data privacy, and PHR type.

## 3. Essential Terminologies and Interoperability Quality Standard

A high-level description of PHR-S FM, HL7 FHIR, HAPI FHIR JAVA RESTful API, clinical vocabularies, and TSD has been captured in Appendix A. A subset of standard functional components in PHR-S FM for this study has been described in Table 2 to maintain the quality of interoperability and PHR implementation standards. In general, many PHR design and development does not directly conform to PHR-S FM; however, they should conform to the PHR-S FM standard for a well-defined functional profile, and the same has been addressed in this study.

## 4. Methods

This section gives a detailed analysis and explanation of a tethered PHR mobile application, (e.g., the health eCoach system [49,50,51,52,53,54]) design and its implementation. We have used HL7 FHIR, HAPI library, SNOMED-CT, and PostgreSQL to ensure a bi-directional communication between the eCoach mobile app and PostgreSQL and used API security methods [55,56] to protect HAPI REST APIs. For the overall design and development, we adopted the established iterative approach. 

In our solution approach, we *first* define the scope of the solution, (e.g., modular design pattern following an object-oriented architecture, protocols, ethical approvals, and data types). *Second*, we elaborate on our adopted structural and semantic interoperability methods, (e.g., HL7 FHIR, SNOMED-CT). *Third*, we discuss interoperability quality standards to design and develop our tethered mobile PHR, (e.g., PHR-S FM). *Forth*, we describe a system architecture where we have deployed PHR services, HL7 FHIR server, and set up the EHR. *Finally*, we discuss semantic and structural interoperability verification methods for our tethered PHR solution. The overall steps to answer the identified research questions are summarized in Box 1.

Box 1 **The steps to answer the identified research questions.**

**(Step-1)** Enable PGHD recording and management through eCoach mobile app.

**(Step-2)** Set up a HAPI FHIR server inside the TSD system to store and access the clinical information.

**(Step-3)** Create a web-based application to define HL7 FHIR profiles for our eCoach prototype system.

**(Step-4)** Implement the RESTful HAPI FHIR server profiles where clinical data is stored as FHIR resources.

**(Step-5)** Create necessary services to support the HAPI FHIR server, enable data and endpoint security, and

**(Step-6)** Enable structural and semantic interoperability across diverse devices (or systems) as FHIR resources with SNOMED-CT integration (in JSON format).

### 4.1. Scope of the Solution

**Figure 1:**
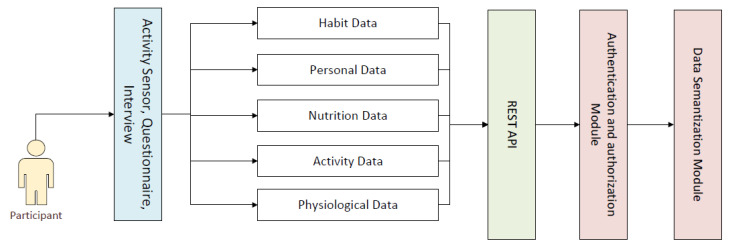
The modules of the eCoach prototype system.

An eCoach system is a multifaceted non-linear system. It consists of loosely coupled electronic components that interact across various feedback loops. A health eCoach system may help to generate automatic and personalized or community-based recommendations based on the insights from personal health and wellness data to achieve healthy lifestyle goals. Our designed and implemented eCoach system has the following modules—a. data collection module, b. security module for REST API authentication and authorization, c. annotation module for data semantization, d. health state monitoring module to analyze the behavioral pattern over time, e. personalized recommendation generation, and f. the recommendation presentation in the eCoach mobile app with a user-centered design (UCD) approach. However, this study focuses on the first three modules. The data collection module (see Figure 1) is responsible for collecting an identified list of personal and person-generated health and wellness data over time, as described in Table 3. The participants must authenticate and authorize themselves to upload their data to the secure TSD system through the eCoach mobile app.

In TSD, data are stored in PostgreSQL as FHIR resources. The data are protected from personal identity disclosure as no individual or national level identifiers are planned to collect from participants following the ethical approval by the Norwegian Centre for Research Data (NSD) guidelines [57]. The data semantization module converts data into a structured JSON format using HL7 FHIR resources and SNOMED-CT vocabularies before storing them in the TSD database. Data processing for demographic statistics, health risk prediction, and decision-making for personalized recommendation generation for healthy lifestyle management are beyond the scope of this study. The visualization of the interface presentation can be further improved using an iterative UCD approach with a dialogue-lab method.

### 4.2. Structural and Semantic Interoperability

According to the Academic Committee for health and architecture in Norway (NUFA), the standards and regulations that have been developed for the Norwegian healthcare system are—OpenEHR, HL7 V3.0 messaging, HL7 CDA R2, IHE XDS.b, Link Data, and HL7 FHIR. The PHR must achieve structural interoperability to seamlessly communicate healthcare data within the PHR and between the PHR and other external resources (such as EHR). 

The main reasons for using HL7 FHIR are—a. FHIR supports the best features of HL7 V2.0, V3.0, and CDA, b. HL7 FHIR does not require any specific language, c. FHIR supports the latest web standards (RESTful architecture) for the developers and implementors, d. FHIR supports both JSON and XML value-set, e. support for RESTful protocol with API-style programming to manage and exchange FHIR resources, f. FHIR supports both international, (e.g., LOINC, SNOMED CT) and a system specified observation codes to formulate “Observation” resource-type, and g. FHIR supports different models of interaction, such as message exchange, document sharing, and text exchange. FHIR terminology module consists of the following resource types [58]: naming system, code system, value sets, elements or coded data types, concept map, and element definition. In this study, we have used SNOMED CT (http://snomed.info/sct (accessed on 10 March 2022)) and its concept details to represent FHIR terminology-related structures and their relationships.

According to the IN.2 of the PHR-S FM framework, a PHR system must support standards-based interoperability for seamless sharing of information between PHRs and other internal or external systems [48]. The same has been addressed in this study. HL7 FHIR stands out as one of the best candidates among HL7 FHIR, ASTM CCR, ISO 13606, HL7 CDA, and CCD to achieve structural interoperability in PHR on the review results of the latest norms, standards, and recommendations. PHRs are more focused on atomic health data objects, such as pulse, blood glucose, lipid profile, and BMI, rather than implementing standards for clinical documents with healthcare data in document-centric approaches. The document-centric policy deals with documents, such as reports, individual records, hospital information, insurance data, and their exchange in XML format between diverse systems, appropriate for EHRs. HL7 FHIR resource profiling improves the data quality and helps experts manage an atomic data entity or a group of data entities or records, (e.g., PHR). Such features of HL7 FHIR make it suitable to design and implement interoperable PHRs compared to the current standards.

Furthermore, the atomic part of HL7 FHIR supports the reusability of data elements. A PHR can target a specific group of individuals, (e.g., only adults) and specify data elements in design and implementation. HL7 FHIR has various resource types that help experts build a generic and PHR-centered profile to assign a meaningful value consisting of semantic vocabularies, such as LOINC and SNOMED-CT, (e.g., vital-sign body weight has a unique SNOMED code 27113001 under Observation resource). The value-set is JSON used in this study to represent the following example FHIR resource types [59]: AllergyIntolerance, Appointment, AppointmentResponse, Observation, Person, Questionnaire, and RiskAssessment (see Appendix G). We collected personal allergy data during the participant’s recruitment (interviewing) as baseline data. Trained health professionals, (e.g., nurses) scheduled an appointment for participant screening and assessing participant’s health condition during recruitment and monthly routine check-up to collect physiological data. Daily activity levels were observed using a Bluetooth-enabled activity tracker (e.g., MOX2-5 activity sensor). As a self-reported questionnaire, nutrition and habit information were collected daily, on alternative days, and weekly. 

The Information Infrastructure (IN) function IN 1.8 of the PHR-S FM framework states that the PHR requires a standard semantic vocabulary or terminology to ensure data consistency and correctness and facilitate data interoperability. Moreover, individuals should have the ability to record and manage their data in PHR (PH.3.1.1), and the data can be both structured and unstructured from heterogeneous sources. Our study uses SNOMED-CT vocabularies to enable semantic interoperability in our PHR design and implementation. SNOMEDs are used as a standard to encode EHR data and capture clinical information. SNOMED-CT has 311,000 concepts, representing 1.3 million relationships between them. A unique conceptual ID identifies them (SCTID) with a unique human-readable, fully specified name. The semantic interpretation of PGHD (see Table 3) has been described in Appendix B with SCTID.

### 4.3. Functional Requirements for PHR as an Interoperability Quality Standard

The architecture, design, and implementation of the eCoach prototype application are based on the functional requirements specified by the PHR-S FM and primarily focus on the semantic and structural integration to manage data in a meaningful way. The focus of this study excluded detailed implementation and analysis of security and privacy, as we have used an established TSD system for API security and data privacy. Additional functions from this study are administration, insurance, medication, and finance. Our focus is to implement a PHR using HL7 FHIR; the designed and implemented eCoach app is compatible with the PHR-S FM rather than a general functional profile. To demonstrate the capabilities and usability of HL7 FHIR, keeping Personal Health management in focus, the selected functions from PHR-S FM, at a detail level, are specified in Box 2.

Box 2 **The selected functionalities from PHR-S FM.**

➢ Identify and Maintain a PHR Account Holder (PH.1.1)
➢ Manage PHR Account Holder Demographics (PH.1.2)
➢ Manage PHR Account Holder Originated Data (PH.2.1)
➢ Manage Historical and Current State Data (PH.2.5)
  ○ Manage Problem Lists (PH.2.5.1), (e.g., chronic conditions)
  ○ Manage Allergy, Intolerance, and Advance Reaction List (PH.2.5.4), (e.g., known list of allergies, irritations)
  ○ Manage Test Results (PH.2.5.3), (e.g., monitoring)
  ○ Manage Medical History (PH.2.5.6), (e.g., chronic conditions in a year)
  ○ Manage Social History (PH.2.5.10), (e.g., education. Employment)
➢ Manage Personal Observations and Care (PH.3.1.1)
➢ Entity Access Control (IN.3.3)
➢ Manage Self-Assessment (PH.6.2)
➢ Manage Interoperability of PHR Account Holder Demographics (S.3.1)

### 4.4. System Architecture

**Figure 2:**
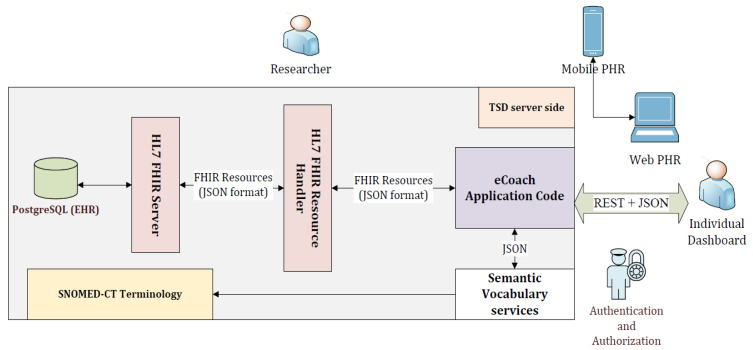
The client-server architectural view of tethered PHR solution with HL7 FHIR and SNOMED-CT.

**Figure 3:**
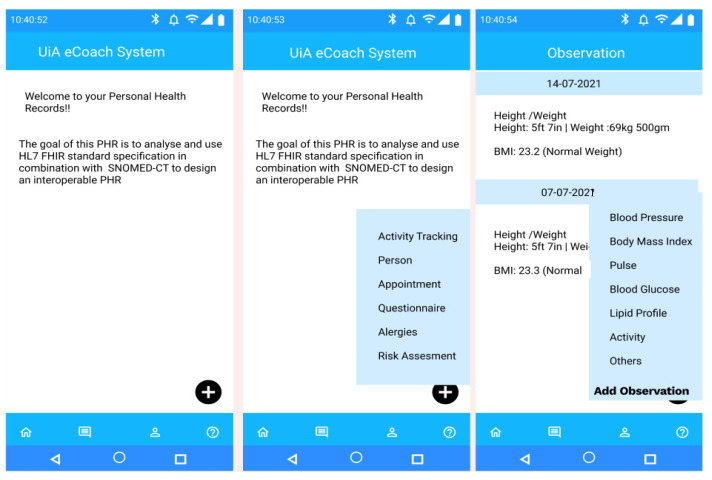
Landing view of the PHR mobile app and its different self-management options.

The architecture consists of two parts: client and server (see the architectural view in Figure 2). The client part consists of mobile PHR, web PHR, and individual (participants, health professionals, and researchers) dashboard. The server includes the eCoach prototype, HL7 FHIR handler and server, PostgreSQL database (EHR), and SNOMED-CT vocabularies hosted inside TSD-based digital infrastructure. The client communicates with the server following the TSD-based authentication and authorization method. All the PGHDs are collected from different sources (activity sensors, questionnaires, and interviewing) through mobile PHR at the client-side and then sent the collected data to the server-side in the JSON format for semantic vocabulary mapping (HL7 FHIR + SNOMED-CT) before getting stored into the PostgreSQL database. Individuals can visualize and update their PGHD through the eCoach system’s PHR menu (see Figure 3), supporting the FHR-S FM functionality PH.1.2, PH.2.1, and PH.2.5. The eCoach application code manages all the logic related to user validation and annotation of incoming JSON into FHIR resource type using SNOMED-CT semantic vocabulary services and sending the JSON to HAPI RESTful service endpoints for data persistence. The FHIR resources are exchanged (in and out) in JSON format. The architecture adopts a modular approach and relies on REST APIs to interact with external components. Therefore, it supports both the mobile and web-based PHRs.

We have developed a digital infrastructure after extending the TSD features with UiA virtual private network (VPN), firewall, and secure socket layer or SSL (HTTPS). The TSD infrastructure supported data capturing from eCoach mobile PHR, stored data in PostgreSQL (or EHR), secured legitimate users’ access, and protected data in the EHR. We have deployed our eCoach prototype system and HAPI FHIR services in the developed infrastructure to execute a formal interoperability testing of tethered PHR solution. TSD uses the Thinlinc remote access protocol based on HTML5 and applies Nginx proxy for two-factor authentication. Thinlinc is a remote desktop framework that supports HTML5 to connect to their Linux machines using a browser instead of a VNC client. Thinlinc utilizes TigerVNC and provides additional user and agent VM management layers, allowing project user groups to be automatically assigned to Thinlinc agents installed on Linux VMs. The Thinlinc infrastructure is composed of Thinlinc agents and Thinlinc masters. The proxy runs Nginx and a custom login protocol. The Thinlinc main server redirects the user after being authenticated through the proxy. The host acts as a proxy, tracking all Thinlinc proxy machines and redirecting users to the correct proxy machine. The user needs to perform an additional login on the actual project VM. Each service deployed inside the TSD platform, (e.g., /v1/<<project number-P1075>>/fhir) is associated with a unique long-term API key ({“api_key” = <key>}). The key is valid for one year and expires automatically. The system administrator orders a new key using the client_id and password (superuser) specific to the application (such as eCoach). The key is just a JWT token to be decoded for verification. The API key is used as a bearer token in the HTTP request header to generate a short-term access token (valid for 30 days) so that the TSD API can authenticate the deployed application. If someone abuses the API, the long-standing API token will be revoked.

### 4.5. Interoperability Verification

Interoperability in the healthcare system can be classified as follows: device interoperability, network interoperability, structural interoperability, semantic interoperability, and platform interoperability [60,61,62,63,64,65]. However, we have focused only on structural and semantic interoperability in this study. For structural interoperability, we have used HL7 FHIR for a uniform representation of PGHDs in JSON format based on different FHIR resource profiles (see Appendix G). We have used semantic vocabularies and SCTIDs of SNOMED-CT medical ontology for semantic interoperability in the FHIR resource profiles (see Appendix B). Furthermore, to establish semantic interoperability, we integrated data from different simulated external sources in the form of a smartphone, desktop, and tablets. PHR data captured through the app has been merged with the data in the EHR to support semantic interoperability. The holistic idea is to accomplish semantic and structural interoperability or integration in a tethered PHR mobile application following a standard guideline and functionalities, (e.g., PHR-S FM) as a quality standard. 

This study has shown a direction to achieve semantic and structural interoperability with HL7 FHIR and SNOMED-CT in bi-directional communication in a tethered PHR application as a PoC study. Therefore, the interoperability verification metrics used for this study, (e.g., PHR-S FM functionalities, HR7 FHIR resource profiling, SNOMED codes) are different from traditional network or device, or platform interoperability verification processes. We have validated metrics such as data loss and unreliable performance during the record fetching from the TSD database or EHR using HL7 FHIR RESTful microservices (HTTP GET) for devices and smartphone apps, and desktop web. To measure the data loss and unreliable performance, we have used Apache open-source software JMeter (V 5.4.1) to generate HTTP requests and capture the outcomes. We set the cycle count value of 5 and took the average data loss and unreliable performance probabilities. The specification of the two devices involved in testing is described in Table 4.

The metric for data loss has been calculated as follows:

$$\mathrm{Data}\;\mathrm{loss}\;(\mathrm{Loss}\%)=\frac{\mathrm{Number}\;\mathrm{of}\;\mathrm{bytes}\;\mathrm{not}\;\mathrm{received}\;\left(n1\right)\;}{\mathrm{Total}\;\mathrm{number}\;\mathrm{of}\;\mathrm{bytes}\;\left(N1\right)}*100$$

The probability of the unreliable performances has been calculated as follows:

$$\mathrm{Probability}\;(P)=\frac{\mathrm{Number}\;\mathrm{of}\;\mathrm{failing}\;\mathrm{cases}\;\left(n2\right)\;}{\mathrm{Total}\;\mathrm{number}\;\mathrm{of}\;\mathrm{cases}\;\mathrm{under}\;\mathrm{consideration}\;\left(N2\right)}$$

## 5. Results

In this study, we describe the eCoach prototype implementation process and the results associated with the interoperability verifications. We developed the eCoach prototype with Java Programming Language following the Object-Oriented Analysis and Design. Appendix C mentions all the used software and corresponding versions for the eCoach prototype implementation. The HAPI-FHIR module has been deployed between the eCoach application. The TSD database stores the Java-based HL7 FHIR resources, extensions, and HAPI core library profiles (see Figure 2). For the interoperability verification, we first performed integration testing between PHR (eCoach app and eCoach web), HAPI FHIR, and EHR (TSD database) at the test server, following the steps mentioned in Appendix D. Then, we deployed the eCoach and HAPI-FHIR services in the developed TSD infrastructure, following the steps mentioned in Appendix E. TSD uses PostgreSQL as its central database server; it has been used as an EHR to store PGHDs. The codes for accessing HAPI FHIR resources in the TSD infrastructure following the authentication and authorization process are described in Appendix F. 

**Figure 4:**
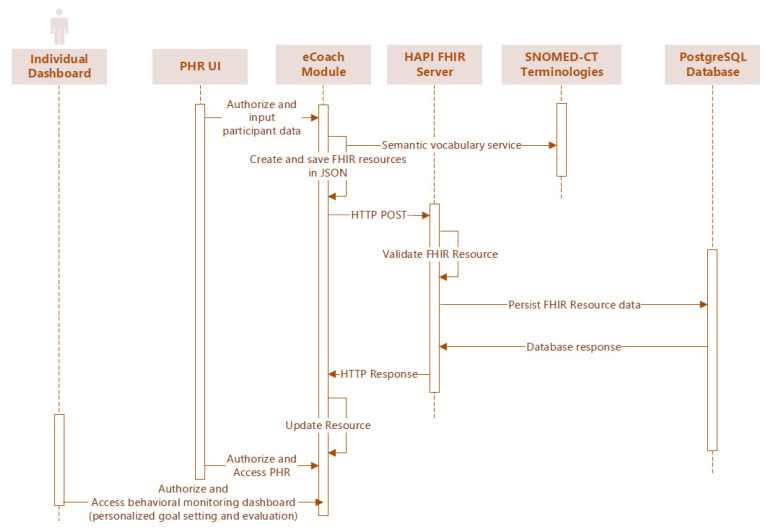
Sequence diagram to synchronize data from PHR (eCoach) with EHR (TSD PostgreSQL).

Our PHR (or eCoach) mobile app collects PGHDs from heterogenous sources and stores them as HL7 FHIR resources in the EHR (or the TSD database) with SNOMED-CT semantic vocabularies in JSON format. The stored resources can be fetched through the HTTP GET method through HAPI FHIR REST endpoints in JSON format. Individuals with PHR mobile app can record and manage their data following the TSD security process. TSD conforms to GDPR and NORMEN guidelines to enable health data security, lawful basis, data transparency, data privacy rights, accountability, and data governance. The data synchronization process between PHR and EHR is depicted in Figure 4 as a sequence diagram.

The landing view of the PHR mobile app (see Figure 3) represents a floating menu at the bottom of the application with the following options—activity tracking (activity data), person (personal data with preferences for personal goal management), appointment (participant’s appointment information with health professionals at the time of initial recruitment and followed by, periodic health assessment), questionnaire (self-reported behavioral data), allergies (food allergy information), and risk assessment (health risk prediction for personalized recommendation generation). The menu options represent a high-level functional overview of the FHIR resource types. The observation interface records observation data in SNOMED-CT terms using the corresponding SCTID. Participants have the flexibility to add more observational data (in context) for a respective date using the “Add Observation” button. For example, they can add the “type of activity” they have performed, (e.g., swimming, cycling). All data are stored in the database as an FHIR resource with SNOMED-CT vocabularies (see Appendix B). 

**Figure 5:**
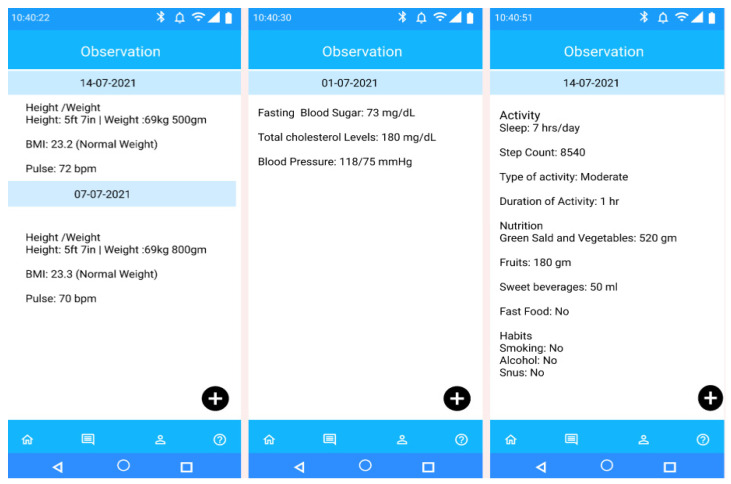
Mobile PHR view of participant’s observational data on different date for self-management as an example of supporting the FHR-S FM functionality (PH.2.5).

Figure 5 depicts the Mobile PHR view of participants’ observational and allergic data in a human-understandable format as an example of supporting the FHR-S FM functionality (PH.2.5). The PHR mobile app has the flexibility to update FHIR resources and process them for personalized health risk assessment. Accurate prediction of health risks may help successful recommendation generation manage personal goals. The individual dashboard enables authorized access to the personalized behavioral monitoring dashboard. The actual JSON structure of the relevant FHIR resources is represented in the Appendix G, following the semantic and structural rules. In Table 5, we have explained our achieved PHR-S FM functionalities, and Table 6 describes the addressed challenges in the implementation of the eCoach prototype as a tethered PHR following the PHR-S FW framework as a standard.

To test the Loss% and the probability of unreliable performances (P) during the record fetching from the TSD database, we selected two HL7 FHIR REST services (HTTP GET) with approximately 363 and 2025 bytes of request bodies, 63.02 and 452.5 Kilobytes of the response bodies, and response times of 190 and 480 milliseconds, respectively, using JMeter. With JMeter “Thread Group” feature, we executed five requests/seconds with an acceptable delay of 200 milliseconds. We then took the average value of Loss% and P. In every pass, we obtained n1 = 0 and n2 = 0, with an error% = 0 for both the request bodies. We repeated the same experiment for a smartphone app and a desktop web and obtained similar results. 

The overall interoperability verification results for this study are summarized as follows—a. devices, (e.g., smartphones, desktop PC) are successfully connected with the eCoach REST endpoints, b. data transfer has been successful between the devices and the eCoach system without any data loss and unreliable performances, c. implementation for semantic representation of PGHD has been successful with SNOMED-CT in JSON format, d. the implementation of data parsing, data transfer, and data visualization algorithms are working correctly, e. handling and representation of data are correct across multiple devices, f. the structural interoperability and semantic interoperability in this PHR (or eCoach system) design and implementation have conformed to the PHR-S FM functional requirements, g. the overall E2E communication between eCoach app and PostgreSQL is secured using TSD system and it follows the PHR-S FM functional requirements, and h. successful resource profiling with HL7 FHIR. 

## 6. Discussion

### 6.1. Principle Findings and Comparing with Existing Outcomes

This study has presented the design and implementation to achieve structural and semantic interoperability in a tethered PHR following a standard guideline specified in the PHR-S FM framework and the same is missing or not reported in the existing studies. The main objective of our mobile PHR (eCoach system) is health data management, self-monitoring, and goal management based on personalized recommendation generation to promote a healthy lifestyle. As described in the Results Section, our eCoach prototype has implemented most of the PHR-S FM functions. We successfully achieved structural interoperability and enhanced data quality with HL7 FHIR resource profiling and semantic interoperability with SNOMED-CT medical vocabularies. The adopted architecture also supports design flexibility with a modular design pattern. 

The achievements of this study are summarized as—a. *Interoperability*: the presented work leverages HL7 FHIR to achieve structural and semantic interoperability in collaboration with SNOMED-CT to record and manage data using FHIR resources in the eCoaching context. PHR data captured through the app has been merged correctly with the data in the EHR, b. *Flexibility*: the presented work uses HL7 resource profiling to collect and share PGHDs. The FHIR resource profiling has improved data quality and correctness with meta-data presentation with web-based technologies. Previous standards, such as ASTM CCR, IEEE 11073, HL7 CDA, have either no support for the profile concept or lack tools or documentation to define them efficiently, c. *Easy to manage*: here, we have used JSON meta-data to exchange data between PHR and EHR. All studies except Saripalle et al. used a document-centered approach to design the PHR. OpenEHR has similar resources to FHIR; however, it supports Archetype Definition Language (ADL), which is more complex than FHIR-supported UML and JSON, d. *Standards*: previous research developed PHR applications without using an established and mature standard. However, this research supports HL7 FHIR, SNOMED-CT, and PHR-S FM to achieve interoperability successfully. Furthermore, this study overcomes the limitations associated with the Indivo model [40] to design and develop PHR, e. *Lightweight protocols*: the HL7 FHIR supports REST APIs and JSON data format to establish the PHR communication with EHR or other sources. REST protocols are lightweight and worldwide accepted standards for data communication over the web. Moreover, JSON parsing is faster than XML parsing, f. *Data integration*: to establish semantic interoperability, we performed successful integration of data from different external sources, and g. *Security*: to establish the interoperability between the PHR and EHR, we verified that data entered from the PHR can be integrated into the EHR, and the data from the EHR can be utilized by the PHR in an effective way, and i. the presented work achieves data security, API security, and privacy using the TSD-based authentication and authorization mechanism. Data security and privacy are lacking in most of the previous studies except for studies conducted by Lee et al., Margheri et al., Hawig et al., Hylock et al., Rohers et al., Mandel et al., and Kyazze et al.

### 6.2. Limitations and Future Scope

In this study, we have consolidated the implementations of PHRs in the research articles and excluded the execution and performance of commercially available PHRs on the internet. We have accomplished a high-level functional comparison of different PHR implementations; instead, a detailed level analysis of which functions are the best. Each PHR has been developed for respective patient populations with specific datasets. Therefore, a generalized functionality is complicated to form. 

The presented prototype does not address functionalities (of PHR-S FM) related to insurance information, medication management, messaging, lab reports, test results, medication lists, and advanced data sharing. We wish to overcome such limitations in the current design and development of PHR in our future studies. We have used HAPI FHIR RESTful microservices, an open-source FHIR API for Java. It might create a problem in legacy systems or enterprise servers where only C#.NET. is supported. A detailed usability study is essential for the developed eCoach prototype as an FHIR mobile application to evaluate the acceptance or credibility of end-users. We will perform a qualitative and quantitative performance evaluation to perform a real-world validation in our future studies. This study has only focused on syntactic or structural and semantic interoperability and excluded other interoperability verification studies, such as network, device, or platform. In our future studies, we will address them. However, even with enumerated limitations, this study will be helpful for the healthcare research community to achieve structural and semantic interoperability in PHRs.

## 7. Conclusions

This study has elaborated the design and development of structural and semantic interoperability in a tethered PHR with HL7 FHIR resources combined with SNOMED-CT vocabularies. Then, we introduce the concept of using PHR-S FM standard guidelines to design and implement a PHR mobile application. The data captured in PHR is constructed as FHIR resources and shared with EHR in JSON format using Restful API services. Furthermore, the TSD system protected the PHR from illegitimate access. This study explains the unique perspective and architecture outline for implementing an interoperable PHR using modern healthcare standards that expose health data as services using APIs and RESTful protocols. Moreover, this study describes HAPI FHIR Services integration with a health monitoring and recommendation generation system, (e.g., health eCoach prototype system) to achieve semantic representation, record, manage, and exchange PGHDs following the international standards, (e.g., HL7 FHIR, SNOMED-CT).

## Figures and Tables

**Table 1:** Summary of the previous work in comparison to our work.

Research Group	Year	Integration Standards	Security and Authentication	Data Privacy	PHR Type
Chatterjee et al. (Our work)	2021	HL7 FHIR, SNOMED, JSON, TSD, PostgreSQL, PHR-S FM	Yes	Yes	Tethered
Hommeaux et al. [26]	2021	FHIR, RDF, ShEX	No	No	Standalone
Gruendner et al. [34]	2021	FHIR, JSON, and PostgreSQL	No	No	Tethered
Gulden et al. [27]	2021	FHIR	No	No	Tethered
Tao et al. [47]	2021	HL7	No	No	Tethered
Zong et al. [36]	2021	HL7 FHIR	No	No	Tethered
Verma et al. [35]	2021	OpenMRS	No	No	Integrated
Lee at al. [41]	2020	FHIR	Yes	Yes	Integrated
Mandl et al. [28]	2020	HL7 FHIR, SMART	No	No	Integrated
Margheri et al. [42]	2020	HL7 FHIR, IHE	Yes	Yes	Integrated
Pfaff et al. [37]	2019	CAMP FHIR	No	No	Tethered
Odigie et al. [44]	2019	SNOMED, FHIR, and CQL	No	No	Tethered
Hawig et al. [46]	2019	FHIR	Yes	Yes	Tethered
Hylock et al. [43]	2019	FHIR	Yes	Yes	Integrated
Zhang et al. [45]	2019	FHIR, LOINC, HPO	No	No	Tethered
Kiourtis et al. [29]	2019	HL7 FHIR	No	No	Tethered
Saripalle et al. [11]	2019	HL7 FHIR, OpenEMR, PHR-S FM, SNOMED, RxNorm	No	No	Tethered
Hussain et al. [30]	2018	HL7 FHIR	No	No	Standalone
Li et al. [38]	2017	HL7 CDA/CCD	No	No	Integrated
Rohers et al. [39]	2017	OpenEHR	Yes	Yes	Integrated
Bloomfield et al. [33]	2016	HL7 FHIR, SMART	No	No	Tethered
Plastiras et al. [31]	2016	HL7 CDA	No	No	Tethered
Mandel et al. [21]	2016	FHIR, SMART	Yes	Yes	Tethered
Kyazze et al. [32]	2014	ASTM CCR	Yes	Yes	Standalone
Cerón et al. [40]	2014	Indivo Model	No	No	NA

**Table 2:** Adopted functions defined in PHR-S FM [11,48].

ID	Function Name	Relevant Tasks for This Study
PH.1	Account holder profile	It helps individuals with guidelines for installation, initialization, enrollment, or operation of their PHR.
PH.3	Wellness preventive medicine and self-care	It helps PHR account holders record and manage their health records from heterogeneous sources in both structured and unstructured formats.
PH.5	Account holder decision support	It helps to PHR account holders receive decisions based on their health conditions.
PH.6	Manage encounters with providers	It helps PHR account holders self-assess some symptoms for which they need to meet with the provider.
S.1	Provider management	It helps PHR account holders schedule appointments and ask health-related questions. Furthermore, it helps import or retrieve data essential to identify a health care provider or health care facility.
S.3	Administrative management	It helps PHR account holders manage account related administrative operations.
IN.1	Health record information management	It helps PHR account holders extract health information, including data aggregation, data exchange, analysis, reporting and printing services.
IN.2	Standard-based interoperability	It supports sharing of information between PHRs and other systems (external and internal), such as EHRs, seamlessly, maintaining interoperability, security, and privacy standards.
IN.3	Security	It helps PHR account holders to facilitate secure data communication between health providers.

**Table 3:** The approved list of personal and person-generated health data.

Data Type	Data
Habit	Smoking, snus, alcohol
Personal	Age, gender, education, contact information, (e.g., mobile, email), income group, social participation status, postcode, preferences
Nutrition	Type of foods and drink intake, amount of food intake of the following types: discretionary, vegetables, fruits, and sweet beverages
Activity	Steps, sleep duration, sleep efficiency, exercise type, (e.g., LPA or low physical activity, MPA or medium physical activity, and VPA or vigorous physical activity), sedentary bouts, standing, and weight bearing
Physiological	Pulse, height, weight, BMI, blood glucose, blood pressure, and lipid profile

**Table 4:** Specifications for the two devices involved in API testing.

Specification	Smartphone App.	Desktop Web.
Operating system	Android	Microsoft Windows
Version	11	10 Enterprise
Processor	Snapdragon 845	Intel Core i5—8265U
RAM	6 GB	16 GB
Storage	128 GB	512 GB

**Table 5:** The achieved functionalities as compared to the identified functionalities [48].

Type of the Function	Type	Achieved?
Basic function	Health record	Yes, they can view their health data in eCoach app from the TSD database.
Basic function	Administrative record	Yes, however, very limited as only managing personal information in the eCoach app functionality has been implemented.
Advanced function	Communication	Yes, individuals can interact with the providers and engineers through the eCoach app.
Advanced function	Appointment management	Yes, individuals can manage appointments with health care providers of periodic health check-up through the eCoach app.
Advanced function	Education	Yes, eCoach app. contains relevant online links for self-education and motivation.
Advanced function	Self-health management	Yes, the main objective of the eCoach app. is to motivate individuals for self-monitoring to achieve healthy lifestyle goal with personalized recommendation generations.
Advanced function	Medication management	Not in the scope
Advanced function	Finance	Not in the scope
Advanced function	Insurance	Not in the scope

**Table 6:** The addressed challenges in the implementation of the PHR [48].

Challenge(s)	Description	How Addressed in This Study?
Interoperability	Capability of PHR to exchange data with other internal or external system.	HL7 FHIR for structural interoperability and SNOMED-CT for semantic interoperability following the PHR-S FM framework.
Security and privacy	Protecting data and personal information in PHR including end-to-end communication.	Using the security and privacy mechanism of the TSD system. TSD conforms to GDPR and NORMEN guidelines to facilitate health data security, lawful basis, data transparency, data privacy rights, accountability, and data governance.
Usability	It is important to assure reliability in using PHR effectively	Using the functions of PHR-S FM framework.
Data quality	It guarantees reliability, accuracy, timeliness, and completeness of the PHR information	With HL7 FHIR resource profiling
Personalization	Capability of PHR to be personalized and altered to individual requirements and preferences.	With personal preference data for goal settings, response type, and interaction type for the tailored recommendation generation. In addition, individuals can edit or view or manage their information without tampering them.

## Data Availability

Not applicable. We used and displayed data of dummy participants. Therefore, no personal identity has been disclosed.

## Abbreviations

PoC	Proof of Concept
EHR	Electronic Health Record
EMR	Electronic Medical Record
PHR	Personal Health Record
PGHD	Personal and Person-Generated-Health (and wellness) Data
FHIR	Fast Healthcare Interoperable Resources
PHR-S FM	Personal Health Record System Functional Model
eCoach	Electronic Coaching
SNOMED-CT	Systematized Nomenclature of Medicine -- Clinical Terms
LOINC	Logical Observation Identifiers Names and Codes
ICD-11	International Classification of Diseases 11th Revision
TSD	Services for Sensitive Data
NUFA	Academic Committee for health and architecture in Norway
NSD	Norsk Senter for Forskningsdata
GDPR	General Data Protection Regulation
HTTP	Hypertext Transfer Protocol
REST	Representational State Transfer
JSON	JavaScript Object Notation
XML	Extensible Markup Language
VPN	Virtual Private Network
UML	Unified Modelling Language
CDA	Clinical Document Architecture
CCR	Continuity of Care Record
CCD	Continuity of Care Document.

## Appendix A

**Table A1:** The high-level descriptions on the used key terminologies.

Key Terms	Description
PHR-S FM	While PHR is the fundamental single and logical personal or patient record, the PHR-S FM [11,48,66,67] acts a standard framework with guidelines, specifications, and a set of functions for PHR implementation and to manage and maintain the records. PHR-S helps to accomplish various purposes on the records, such as health decisions, administrative (e.g., provider and financial management), health education, research, wellness, and the determination of public health. It acts as a gold standard for PHR system development and enables consistent expression of functionality, flexible innovation, and product differentiation. PHR-S supports three different types of functions, such as personal health (PH.1 Account holder profile, PH.2 Manage historical clinical data and current state data, PH.3 Wellness, preventive medicine and self-care, PH.4 Manage health education, PH.5 Account holder decision support, and PH.6 Manage encounters with providers), supportive (S.1 Provider management, S.2 Financial management, S.3 Administrative management, and S.4 Other resource management), and information infrastructure (IN.1 Health record information management, IN.2 Standards-based interoperability, IN.3 Security, and IN.4 Auditable records). The functions in PHR-S enable individuals to record and manage their personal health data (PGHD). We have captured some PHR-S functions in Table 2 which are relevant for this study.
TSD	TSD [68] is an IT platform for collecting, storing, checking, and providing sensitive information in compliance with Norwegian protection guidelines [69,70]. TSD is used for research, clinical study, and commercial research. TSD was created and worked by the University of Oslo (UiO) and is part of NorStore, which is the common foundation for processing logical information and capacity. The creation of TSD is divided into two phases: the first phase is the 2009–2011 commitment, and the second phase is 2012–2014. Here, project planning and advancement start from an absolute starting point, and the focus is determined by the use case. All services and data are protected inside the TSD to prevent illegal external access. TSD provides services such as-client registration (register, confirm, obtain API key and password reset), use access token for authentication and authorization, JSON file import and export (simple file upload and download, recoverable file upload and download), resume uploads and downloads, four different types of access tokens, used for basic authorization and TSD authorization services (survey_import, survey_export, survey_admin and survey_member), filter queries, and encryption of JSON and file data (Base64 encoding).
HL7 FHIR	HL7 provides a framework and related standards to exchange, integrate, share, and retrieve digital health information. Such standards define how data is packaged and transmitted from one system to another with seamless integration between systems. The HL7 standard supports clinical practice and the management, provision, and evaluation of health services and is recognized as the most used standard in the world [71]. HL7 V2.0 was created in 1988 with the following message structure—message (message type, trigger event, and abstract message syntax table), segment, field, data type, and vocabulary. HL7 V3.0 replaced HL7 V2.0 in 1992 for stringent, model-based approach [72]. FHIR, a next-generation messaging standards framework, can be considered an HL7 V4.0 [30]. FHIR combines the best features of HL7 products to leverage the latest web standards focusing on implementation ability. FHIR is a collection of modular components called resources, created, and managed by HL7. Resources can easily be assembled into working healthcare systems that solve real-world clinical and administrative problems. FHIR resources can be categorized among the following groups [73]—foundation, base, clinical, financial, and specialized. In FHIR, PGHDs can be an accumulation of relevant resources (see Appendix A). FHIR is suitable for various contexts, such as smartphone apps, cloud communications, PHR-based data sharing, and communication in large institutional healthcare providers. Resources are referenced by uniform resource identifiers (URIs) and exchanged between systems using the web approach (RESTful API) as a bundle of resources (messages and documents) following the client-server topology and protocols utilized in the World Wide Web (WWW) [30,72]. FHIR supports both the XML and JSON parsers for object annotation and information exchange [26]. The lightweight nature of JSON has helped FHIR to adopt RESTful replacement for IHE-XDS protocol based on simple object access protocol (SOAP)/XML with IHE-MHD for mobile access to health documents [30].
HAPI FHIR REST API	HL7 FHIR is a specification; however, the HAPI library is a java-based open-source implementation of the HL7 FHIR specification. The University Health Network developed the library to add FHIR competencies to existing healthcare applications. It has been implemented in over 800 FHIR projects, and 120 contributors have been involved [26]. The HAPI library defines classes for each FHIR resource, data type, and value set defined by the FHIR specification [11] and consists of the following core modules—a core library module, a structure model, a client framework, and a validation module to validate FHIR resource instances against FHIR profiles [26]. The HAPI library provides the FHIR server and client. The server is a traditional web server that supports the REST protocol, and the client can generate REST requests and send them to the server using HTTP implementation [11,23]. The library also promotes verification of modeled FHIR resources to ensure that the resources meet specifications [11].
Clinical vocabularies	Medical vocabularies, such as LOINC, SNOMED-CT [74] are international standards to identify health measurements, observations, and documents. SNOMED-CT, International Classification of Diseases (ICD-11), Unified Medical Dictionary System (UMLS Semantic Network), Anatomical Basic Model (FMA), OpenEHR, Gene Ontology (GO), DOLCE, basic formal ontology, Cyc’s upper-level ontology, Sowa’s top-level ontology, GALEN’s top-level, and LOINC are several biomedical ontologies (or clinical terminologies) that have been introduced to offer semantic interoperability and complete knowledge related to the biological and medical field [74]. Most laboratories and clinical systems use the HL7 (V. 2) protocol to send data. In HL7 messages, the LOINC code represents the “question” of the laboratory test or experiment, and the SNOMED CT code means the “answer”. This study reused the SNOMED-CT ontology to model health based on health data with FHIR [74]. SNOMED-CT was designed in 1965 as a controlled medical vocabulary licensed and supported by the international health term SDO [74]. It is an organized, comprehensive, and multilingual list of various standard clinical terms defined by unique codes (ICD) for easy and automatic interpretation and representation of clinical phrases. It covers a wide range of diseases and findings (what has been observed?), procedures (what has been done?), events (what happened?), substances/drugs (what has been consumed or administered?), and any clinical data. It provides a shared language that enables a reliable way to index, store, retrieve, and accumulate clinical data across healthcare domains and nursing sites. It is a complete and scientifically validated multilingual clinical term that provides a consistent representation of clinical contents in EHRs and clarity for clinical documents and reports [74,75]. Integration of SNOMED-CT into FHIR can harness the rich representability (e.g., unambiguity, structured, cohort, easy decision making) of clinical terminologies for semantic interoperability during the exchange of FHIR resources (e.g., PGHD, PHR) between systems [76]. The power of FHIR in SNOMED-CT may produce the best health information model [77,78].

## Appendix B

**Table A2:** Semantic interpretation of personal and person-generated health data with SNOMED.

Data *	Type	SCTID	FHIR Resource
Smoking	Habit	229819007	Questionnaire
Snus	Habit	713914004	Questionnaire
Alcohol	Habit	897148007	Questionnaire
Medical Record Number	Personal	398225001	Person
Age	Personal	424144002	Person
Gender	Personal	263495000	Person
Education	Personal	276031006	Person
Mobile	Personal	428481002	Person
Email	Personal	424966008	Person
Income	Personal	224167002	Person
Social	Personal	699089001	Person
General Sleep duration	Personal	248263006	Person
Postcode	Personal	184102003	Person
Fast food	Nutrition	230112002	Questionnaire
Food allergy	Nutrition	414285001	AllergyIntolerance
Vegetable	Nutrition	226448008	Questionnaire
Salad	Nutrition	227927005	Questionnaire
Fruit	Nutrition	72511004	Questionnaire
Sweet beverages	Nutrition	818989004	Questionnaire
Activity	Activity	68130003	Questionnaire
Type of activity **	Activity	257733005	Observation
Type of posture ***	Activity	363855006	Observation
Sleep	Activity	258158006	Observation
Duration (LPA, MPA, VPA, Weight bearing, standing, sedentary)	Activity	103335007	Observation
Pulse	Physiological	8499008	Observation
Height	Physiological	50373000	Questionnaire
Weight	Physiological	64305001	Questionnaire
BMI	Physiological	60621009	Observation
Blood glucose	Physiological	365812005	Observation
Lipid profile	Physiological	365793008	Observation
Blood pressure	Physiological	75367002	Observation
Systolic blood pressure	Physiological	271649006	Observation
diastolic blood pressure	Physiological	271650006	Observation
Waist-hip ratio	Physiological	248367009	Observation
General practice	Personal	394814009	Appointment
Gelatin	Nutrition	373531009	Allergy
Gelatin allergenic extract Injectable Product	Nutrition	64896002	Allergy
Anaphylactic reaction	Nutrition	39579001	Allergy
Subcutaneous route	Nutrition	34206005	Allergy
Urticaria	Nutrition	64305001	Allergy

* All data used in this table are in accordance with our PoC study. The underlying data are modifiable complying with project requirements. ** Type of activity can be of following three types—“low” or low physical activity (LPA), “moderate” or medium physical activity (MPA), and “high” or vigorous physical activity (VPA) (in accordance with standard wearable activity devices, such as Fitbit, MOX2-5). *** Type of posture can be of following three types—sedentary, weight bearing, and standing (in accordance with standard wearable activity devices, such as Fitbit, MOX2-5).

## Appendix C

**Table A3:** Used software and their versions for this implementation.

Software	Version	Purpose
Java Development Toolkit	13	To compile java codes for system development
Spring Tool Suite	4	To write java codes in SpringBoot framework
SpringBoot	2.2.4	A framework to write codes for eCoach system
Apache Tomcat	9.0.3	A web server to deploy web archive file
Docker Desktop	3.5.2	A container to deploy eCoach and HAPI FHIR
Figma	-	For initial prototyping of eCoach App’s PHR
Microsoft Visio	2019	To prepare diagrams
PostgreSQL	13	To store HL7 FHIR JSON data
PgAdmin4	5.4	To manage PostgreSQL from UI console
VMWare Horizon	-	To access TSD’s secure RedHat 8 VM
Postman	7.0	To test eCoach and TSD REST services with HTTP methods
Mockito	3.10	For unit testing of application modules
JMeter	5.4.1	For capturing data loss and unreliable performance probabilities

## Appendix D

Box A1 Prerequisites to deploy FHIR into the test server.

➢ Setup the working environment with JDK 8.0+ version and Apache-Maven build tool (V 3.X)
➢ Download the HAPI-FHIR starter project from the GitHub link—https://github.com/hapifhir/hapi-fhir-jpaserver-starter (accessed on 10 March 2022).
➢ Install the latest version of Apache-tomcat webserver (e.g., V9.0.3).
➢ Create a database namely “ecoach_fhir” in PostgreSQL (e.g., V13.0)
➢ Configure HAPI-FHIR to PostgreSQL database in the environment property file.
➢ Compiling and installing HAPI FHIR →generate ROOT.war file.
➢ Deploy the web archive file (war) into the tomcat webserver.

## Appendix E

Box A2 Deploying HAPI-FHIR into the developed digital infrastructure with TSD integration.

➢ Install docker desktop in the local PC or laptop.
➢ Download the HAPI-FHIR starter project from the GitHub link—https://github.com/hapifhir/hapi-fhir-jpaserver-starter (accessed on 10 March 2022).
➢ Create a database namely “ecoach_fhir” in PostgreSQL (e.g., V13.0) at TSD side.
➢ Configure HAPI-FHIR to PostgreSQL database in the environment property file.
➢ Compiling and installing HAPI FHIR→create Dockerfile with OpenJDK:12-alpine→generate fhir.tar docker image file.
➢ Upload the container image tarball (ecoach:fhir) into TSD project via the TSD Data Portal (https://data.tsd.usit.no/).
➢ Load the docker image fhir.tar file (docker load -i fhir.tar) in TSD.
➢ Run the image file (docker run -d 8080:8080 ecoach:fhir) in TSD.

## Appendix F

Box A3 Necessary Java code-snippet to authenticate and authorize at TSD.
**2-factor Authentication with credentials and the generation of access token** **—**
  public static void authentication () throws Exception { URL url = new URL (https://api.tsd.usit.no/v1/p1075/auth/tsd/token?type=fhir-generic (accessed on 10 March 2022)); HttpURLConnection http = (HttpURLConnection)url.openConnection(); http.setRequestMethod(“POST”); http.setDoOutput(true); http.setRequestProperty(“Accept”, “application/json”); http.setRequestProperty(“Authorization”, “Bearer **<<api_key provided by TSD>>**”); http.setRequestProperty(“Content-Type”, “application/json”); String data = “{\”user_name\”:\”p1075-XXXX\”, \”otp\”:\”421847\”, \”password\”:\”XXXX\” } “; byte[] out = data.getBytes(StandardCharsets.UTF_8); OutputStream stream = http.getOutputStream();  stream.write(out);      stream.flush(); BufferedReader in = new BufferedReader(new InputStreamReader(http.getInputStream())); String inputLine; while ((inputLine = in.readLine()) != null) {        logger.info(inputLine); **//access token generation** **for authorization** } in.close();         logger.info(http.getResponseCode() + “ “ + http.getResponseMessage());  **//200 OK** http.disconnect();               }  

**Authorization with access token** **—**
  public static void authorize () throws Exception { URL url = new URL(“https://api.tsd.usit.no/v1/<<projectId>>/fhir/**<<FHIR Resource Type>>**”); HttpURLConnection http = (HttpURLConnection)url.openConnection(); http.setRequestMethod(“GET”); http.setDoOutput(true); http.setRequestProperty(“Accept”, “application/json”); http.setRequestProperty(“Authorization”, “Bearer **<<access token>>**”); http.setRequestProperty(“Content-Type”, “application/json”); BufferedReader in = new BufferedReader(new InputStreamReader(http.getInputStream())); String inputLine; while ((inputLine = in.readLine()) != null) {        logger.info(inputLine); } in.close(); logger.info(http.getResponseCode() + “ “ + http.getResponseMessage());        **//200 OK** http.disconnect(); }

## Appendix G

In this section, we have described relevant FHIR resources to capture, store and exchange a dummy participant’s personal and person-generated health data in JSON meta-data format.

#1 – FHIR Resource type: Person

{

  “resourceType”: “Person”,

  “id”: “UiA101”,

  “text”: {

    “status”: “generated”,

    “div”: ““

  },

  “identifier”: [

    {

      “use”: “usual”,

      “type”: {

        “coding”: [

          {

            “system”: “http://snomed.info/sct”,

            “code”: “398225001”

          }

        ]

      },

      “system”: “eCoachUiA”,

      “value”: “101”,

      “period”: {

        “start”: “2021-05-06”

      },

      “assigner”: {

        “display”: “eCoachUiA”

      }

    }

  ],

  “name”: [

    {

      “use”: “official”,

      “family”: “Dave”,

      “given”: [

        “Peter”,

        “James”

      ]

    },

    {

      “use”: “usual”,

      “given”: [

        “Jim”

      ]

    }

  ],

  “telecom”: [

    {

      “use”: “home”

    },

    {

      “system”: “mobile”,

      “value”: “(47) 1234 5678”,

      “use”: “personal”

    },

    {

      “system”: “email”,

      “value”: “Jamesm@example.org”,

      “use”: “home”

    }

  ],

  “gender”: “male”,

  “age”: “25”,

  “education”: “Masters”,

  “income”: “Middle class”,

  “social”: “Yes”,

  “general sleep duration”: “7 hrs.”

  “address”: [

    {

      “use”: “home”,

      “line”: [

        “H0101 Storgata 1A”

      ],

      “city”: “Grimstad”,

      “state”: “Aust-Agder”,

      “postalCode”: “4876”

    }

  ],

  “active”: true,

  “link”: [

    {

      “target”: {

        “reference”: “RelatedPerson/peter”,

        “display”: “Peter Dave”

      }

    },

    {

      “target”: {

        “reference”: “Patient/example”,

        “display”: “Peter Dave”

      }

    }

  ]

}

#2 – FHIR Resource type: Appointment

{

  “resourceType”: “Appointment”,

  “id”: “UIA101”,

  “text”: {

    “status”: “generated”,

    “div”: ““

  },

  “status”: “booked”,

  “serviceCategory”: [

    {

      “coding”: [

        {

          “system”: “http://example.org/service-category”,

          “code”: “gp”,

          “display”: “General Practice”

        }

      ]

    }

  ],

  “serviceType”: [

    {

      “coding”: [

        {

          “code”: “52”,

          “display”: “General Discussion”

        }

      ]

    }

  ],

  “specialty”: [

    {

      “coding”: [

        {

          “system”: “http://snomed.info/sct”,

          “code”: “394814009”,

          “display”: “General practice”

        }

      ]

    }

  ],

  “appointmentType”: {

    “coding”: [

      {

        “system”: “http://terminology.hl7.org/CodeSystem/v2-0276”,

        “code”: “FOLLOWUP”,

        “display”: “A follow up visit from a previous appointment”

      }

    ]

  },

  “reasonReference”: [

    {

      “reference”: “Condition/example”,

      “display”: “High Glycemic Response”

    }

  ],

  “priority”: 5,

  “description”: “Discussion on the results of your recent Glycemic response”,

  “start”: “2021-07-10T09:00:00Z”,

  “end”: “2021-07-10T11:00:00Z”,

  “created”: “2021-07-01”,

  “comment”: “Blood Sugar.”,

  “basedOn”: [

    {

      “reference”: “ServiceRequest/bloodsugar”

    }

  ],

  “participant”: [

    {

      “actor”: {

        “reference”: “Patient/example”,

        “display”: “Peter Dave”

      },

      “required”: “required”,

      “status”: “accepted”

    },

    {

      “type”: [

        {

          “coding”: [

            {

              “system”: “http://terminology.hl7.org/CodeSystem/v3-ParticipationType”,

              “code”: “ATND”

            }

          ]

        }

      ],

      “actor”: {

        “reference”: “Practitioner/example”,

        “display”: “Adam Cox (Nurse)”

      },

      “required”: “required”,

      “status”: “accepted”

    },

    {

      “actor”: {

        “reference”: “Location/1”,

        “display”: “UiA i4Helse, second floor”

      },

      “required”: “required”,

      “status”: “accepted”

    }

  ]

}

#3 – FHIR Resource type: AppointmentResponse

{

  “resourceType”: “AppointmentResponse”,

  “id”: “UIA101”,

  “text”: {

    “status”: “generated”,

    “div”: ““

  },

  “appointment”: {

    “reference”: “Appointment/example”,

    “display”: “Blood glucose results discussion”

  },

  “actor”: {

    “reference”: “Patient/example”,

    “display”: “Peter Dave”

  },

  “participantStatus”: “accepted”

}

#4 – FHIR Resource type: AllergyIntolerance

{

  “resourceType”: “AllergyIntolerance”,

  “id”: “UIA101”,

  “text”: {

    “status”: “generated”,

    “div”: ““

  },

  “identifier”: [

    {

      “system”: “http://acme.com/ids/patients/risks”,

      “value”: “49476534”

    }

  ],

  “clinicalStatus”: {

    “coding”: [

      {

        “system”: “http://terminology.hl7.org/CodeSystem/allergyintolerance-clinical”,

        “code”: “active”,

        “display”: “Active”

      }

    ]

  },

  “verificationStatus”: {

    “coding”: [

      {

        “system”: “http://terminology.hl7.org/CodeSystem/allergyintolerance-verification”,

        “code”: “confirmed”,

        “display”: “Confirmed”

      }

    ]

  },

  “type”: “allergy”,

  “category”: [

    “food”

  ],

  “criticality”: “high”,

  “code”: {

    “coding”: [

      {

        “system”: “http://snomed.info/sct”,

        “code”: “373531009”,

        “display”: “Gelatin”

      }

    ]

  },

  “patient”: {

    “reference”: “Patient/example”

  },

  “onsetDateTime”: “2021”,

  “recordedDate”: “2021-07-10T14:58:00+11:00”,

  “recorder”: {

    “reference”: “Practitioner/example”

  },

  “asserter”: {

    “reference”: “Patient/example”

  },

  “lastOccurrence”: “2020-06”,

  “note”: [

    {

      “text”: “The criticality is high becasue of the observed reaction with Gelatin.”

    }

  ],

  “reaction”: [

    {

      “substance”: {

        “coding”: [

          {

            “system”: “http://www.nlm.nih.gov/research/umls/rxnorm”,

            “code”: “1160593”,

            “display”: “cashew nut allergenic extract Injectable Product”

          }

        ]

      },

      “manifestation”: [

        {

          “coding”: [

            {

              “system”: “http://snomed.info/sct”,

              “code”: “39579001”,

              “display”: “Anaphylactic reaction”

            }

          ]

        }

      ],

      “description”: “Challenge Protocol. Severe reaction to subcutaneous cashew extract. Epinephrine administered”,

      “onset”: “2012-06-12”,

      “severity”: “severe”,

      “exposureRoute”: {

        “coding”: [

          {

            “system”: “http://snomed.info/sct”,

            “code”: “34206005”,

            “display”: “Subcutaneous route”

          }

        ]

      }

    },

    {

      “manifestation”: [

        {

          “coding”: [

            {

              “system”: “http://snomed.info/sct”,

              “code”: “64305001”,

              “display”: “Urticaria”

            }

{

  “resourceType”: “AllergyIntolerance”,

  “id”: “example”,

  “text”: {

    “status”: “generated”,

    “div”: ““

  },

  “identifier”: [

    {

      “system”: “http://acme.com/ids/patients/risks”,

      “value”: “49476534”

    }

  ],

  “clinicalStatus”: {

    “coding”: [

      {

        “system”: “http://terminology.hl7.org/CodeSystem/allergyintolerance-clinical”,

        “code”: “active”,

        “display”: “Active”

      }

    ]

  },

  “verificationStatus”: {

    “coding”: [

      {

        “system”: “http://terminology.hl7.org/CodeSystem/allergyintolerance-verification”,

        “code”: “confirmed”,

        “display”: “Confirmed”

      }

    ]

  },

  “type”: “allergy”,

  “category”: [

    “food”

  ],

  “criticality”: “high”,

  “code”: {

    “coding”: [

      {

        “system”: “http://snomed.info/sct”,

        “code”: “373531009”,

        “display”: “Gelatin”

      }

    ]

  },

  “patient”: {

    “reference”: “Patient/example”

  },

  “onsetDateTime”: “2021”,

  “recordedDate”: “2021-07-10T14:58:00+11:00”,

  “recorder”: {

    “reference”: “Practitioner/example”

  },

  “asserter”: {

    “reference”: “Patient/example”

  },

  “lastOccurrence”: “2020-06”,

  “note”: [

    {

      “text”: “The criticality is high becasue of the observed reaction with Gelatin.”

    }

  ],

  “reaction”: [

    {

      “substance”: {

        “coding”: [

          {

            “system”: “http://snomed.info/sct”,

            “code”: “64896002”,

            “display”: “Gelatin allergenic extract Injectable Product”

          }

        ]

      },

      “manifestation”: [

        {

          “coding”: [

            {

              “system”: “http://snomed.info/sct”,

              “code”: “39579001”,

              “display”: “Anaphylactic reaction”

            }

          ]

        }

      ],

      “description”: “Challenge Protocol.”,

      “onset”: “2021-07-10”,

      “severity”: “severe”,

      “exposureRoute”: {

        “coding”: [

          {

            “system”: “http://snomed.info/sct”,

            “code”: “34206005”,

            “display”: “Subcutaneous route”

          }

        ]

      }

    },

    {

      “manifestation”: [

        {

          “coding”: [

            {

              “system”: “http://snomed.info/sct”,

              “code”: “64305001”,

              “display”: “Urticaria”

            }

          ]

        }

      ],

      “onset”: “2021”,

      “severity”: “moderate”,

      “note”: [

        {

          “text”: “The patient reports that the onset of urticaria was within 20 minutes of taking Gelatin.”

        }

      ]

    }

  ]

}

#5–FHIR Resource type: Observation

Weight

{

  “resourceType”: “Observation”,

  “id”: “UIA-101”,

  “text”: {

    “status”: “generated”,

    “div”: ““

  },

  “status”: “final”,

  “category”: [

    {

      “coding”: [

        {

          “system”: “http://terminology.hl7.org/CodeSystem/observation-category”,

          “code”: “vital-signs”,

          “display”: “Vital Signs”

        }

      ]

    }

  ],

  “code”: {

    “coding”: [      

      {

        “system”: “http://snomed.info/sct”,

        “code”: “27113001”,

        “display”: “Body weight”

      }

    ]

  },

  “subject”: {

    “reference”: “Patient/example”

  },

  “encounter”: {

    “reference”: “Encounter/example”

  },

  “effectiveDateTime”: “2021-07-10”,

  “valueQuantity”: {

    “value”: 170,

    “unit”: “lbs”,

    “system”: “http://unitsofmeasure.org”,

    “code”: “[lb_av]”

  }

}

Blood Pressure

{

  “resourceType”: “Observation”,

  “id”: “UIA101”,

  “meta”: {

    “profile”: [

      “http://hl7.org/fhir/StructureDefinition/vitalsigns”

    ]

  },

  “text”: {

    “status”: “generated”,

    “div”: ““

  },

  “status”: “final”,

  “category”: [

    {

      “coding”: [

        {

          “system”: “http://terminology.hl7.org/CodeSystem/observation-category”,

          “code”: “vital-signs”,

          “display”: “Vital Signs”

        }

      ]

    }

  ],

  “code”: {

    “coding”: [

      {

        “system”: “http://snomed.info/sct”,

        “code”: “75367002”,

        “display”: “Blood pressure”

      }

    ],

    “text”: “Blood pressure systolic & diastolic”

  },

  “subject”: {

    “reference”: “Patient/example”

  },

  “effectiveDateTime”: “2021-07-10”,

  “performer”: [

    {

      “reference”: “Practitioner/example”

    }

  ],

  “interpretation”: [

    {

      “coding”: [

        {

          “system”: “http://terminology.hl7.org/CodeSystem/v3-ObservationInterpretation”,

          “code”: “L”,

          “display”: “low”

        }

      ],

      “text”: “Below low normal”

    }

  ],

  “bodySite”: {

    “coding”: [

      {

        “system”: “http://snomed.info/sct”,

        “code”: “368209003”,

        “display”: “Right arm”

      }

    ]

  },

  “component”: [

    {

      “code”: {

        “coding”: [

          {

            “system”: “http://snomed.info/sct”,

            “code”: “271649006”,

            “display”: “Systolic blood pressure”

          }

        ]

      },

      “valueQuantity”: {

        “value”: 109,

        “unit”: “mmHg”,

        “system”: “http://unitsofmeasure.org”,

        “code”: “mm[Hg]”

      },

      “interpretation”: [

        {

          “coding”: [

            {

              “system”: “http://terminology.hl7.org/CodeSystem/v3-ObservationInterpretation”,

              “code”: “N”,

              “display”: “normal”

            }

          ],

          “text”: “Normal”

        }

      ]

    },

    {

      “code”: {

        “coding”: [

          {

            “system”: “http://snomed.info/sct”,

            “code”: “271650006”,

            “display”: “Diastolic blood pressure”

          }

        ]

      },

      “valueQuantity”: {

        “value”: 63,

        “unit”: “mmHg”,

        “system”: “http://unitsofmeasure.org”,

        “code”: “mm[Hg]”

      },

      “interpretation”: [

        {

          “coding”: [

            {

              “system”: “http://terminology.hl7.org/CodeSystem/v3-ObservationInterpretation”,

              “code”: “L”,

              “display”: “low”

            }

          ],

          “text”: “Below low normal”

        }

      ]

    }

  ]

}

BMI

{

  “resourceType”: “Observation”,

  “id”: “UIA101”,

  “text”: {

    “status”: “generated”,

    “div”: ““

  },

  “contained”: [

    {

      “resourceType”: “Observation”,

      “id”: “height”,

      “name”: {

        “coding”: [

          {

            “system”: “http://snomed.info/sct”,

            “code”: “50373000”,

            “display”: “Height”

          }

        ]

      },

      “valueQuantity”: {

        “value”: 180,

        “units”: “centimeter”,

        “system”: “http://snomed.info/sct”,

        “code”: “258672001”

      },

      “status”: “final”,

      “reliability”: “ok”

    },

    {

      “resourceType”: “Observation”,

      “id”: “weight”,

      “name”: {

        “coding”: [

          {

            “system”: “http://snomed.info/sct”,

            “code”: “64305001”,

            “display”: “Weight”

          }

        ]

      },

      “valueQuantity”: {

        “value”: 104.2,

        “units”: “kilogram”,

        “system”: “http://snomed.info/sct”,

        “code”: “258683005”

      },

      “status”: “final”,

      “reliability”: “ok”

    }

  ],

  “name”: {

    “coding”: [

      {

        “system”: “http://snomed.info/sct”,

        “code”: “60621009”,

        “display”: “Body mass index”

      },

      {

        “system”: “http://loinc.org”,

        “code”: “39156-5”,

        “display”: “Body mass index (BMI) [Ratio]”

      }

    ],

    “text”: “BMI”

  },

  “valueQuantity”: {

    “value”: 32.1

  },

  “interpretation”: {

    “coding”: [

      {

        “system”: “http://snomed.info/sct”,

        “code”: “75540009”,

        “display”: “High”

      },

      {

        “system”: “http://hl7.org/fhir/v2/0078”,

        “code”: “H”

      }

    ]

  },

  “issued”: “2021-07-10T13:27:00+01:00”,

  “status”: “final”,

  “reliability”: “ok”,

  “bodySite”: {

    “coding”: [

      {

        “system”: “http://snomed.info/sct”,

        “code”: “38266002”,

        “display”: “Entire body as a whole”

      }

    ]

  },

  “method”: {

    “coding”: [

      {

        “system”: “http://snomed.info/sct”,

        “code”: “122869004”,

        “display”: “Measurement-action”

      }

    ]

  },

  “subject”: {

    “reference”: “Patient/f201”,

    “display”: “Roel”

  },

  “performer”: [

    {

      “reference”: “Practitioner/f201”

    }

  ],

  “referenceRange”: [

    {

      “high”: {

        “value”: 20

      },

      “meaning”: {

        “coding”: [

          {

            “system”: “http://snomed.info/sct”,

            “code”: “310252000”,

            “display”: “Low BMI”

          }

        ]

      }

    },

    {

      “low”: {

        “value”: 20

      },

      “high”: {

        “value”: 25

      },

      “meaning”: {

        “coding”: [

          {

            “system”: “http://snomed.info/sct”,

            “code”: “412768003”,

            “display”: “Normal BMI”

          }

        ]

      }

    },

    {

      “low”: {

        “value”: 25

      },

      “high”: {

        “value”: 30

      },

      “meaning”: {

        “coding”: [

          {

            “system”: “http://snomed.info/sct”,

            “code”: “162863004”,

            “display”: “Overweight”

          }

        ]

      }

    },

    {

      “low”: {

        “value”: 30

      },

      “high”: {

        “value”: 40

      },

      “meaning”: {

        “coding”: [

          {

            “system”: “http://snomed.info/sct”,

            “code”: “162864005”,

            “display”: “Obesity”

          }

        ]

      }

    },

    {

      “low”: {

        “value”: 40

      },

      “meaning”: {

        “coding”: [

          {

            “system”: “http://snomed.info/sct”,

            “code”: “162864005”,

            “display”: “Severe obesity”

          }

        ]

      }

    }

  ],

  “related”: [

    {

      “type”: “derived-from”,

      “target”: {

        “reference”: “#length”

      }

    },

    {

      “type”: “derived-from”,

      “target”: {

        “reference”: “#weight”

      }

    }

  ]

}

Blood Glucose

{

  “resourceType”: “Observation”,

  “id”: “UIA101”,

  “text”: {

    “status”: “generated”,

    “div”: ““

  },

  “name”: {

    “coding”: [

      {

        “system”: “http://snomed.info/sct”,

        “code”: “365812005”,

        “display”: “Glucose in Blood”

      }

    ]

  },

  “valueQuantity”: {

    “value”: 6.2,

    “units”: “mmol/l”,

    “system”: “http://unitsofmeasure.org”,

    “code”: “mmol/l”

  },

  “interpretation”: {

    “coding”: [

      {

        “system”: “http://hl7.org/fhir/v2/0078”,

        “code”: “A”,

        “display”: “abnormal”

      }

    ]

  },

  “appliesPeriod”: {

    “start”: “2021-07-10T09:30:10+01:00”,

    “end”: “2021-07-10T09:30:10+01:00”

  },

  “issued”: “2021-07-06T15:30:10+01:00”,

  “status”: “final”,

  “reliability”: “ok”,

  “bodySite”: {

    “coding”: [

      {

        “system”: “http://snomed.info/sct”,

        “code”: “308046002”,

        “display”: “Superficial forearm vein”

      }

    ]

  },

  “method”: {

    “coding”: [

      {

        “system”: “http://snomed.info/sct”,

        “code”: “120220003”,

        “display”: “Injection to forearm”

      }

    ]

  },

  “identifier”: {

    “use”: “official”,

    “system”: “http://www.bmc.nl/zorgportal/identifiers/observations”,

    “value”: “6323”

  },

  “subject”: {

    “reference”: “Patient/example”,

    “display”: “Peter Dave”

  },

  “performer”: [

    {

      “reference”: “Practitioner/example”,

      “display”: “A. Goodfellow”

    }

  ],

  “referenceRange”: [

    {

      “low”: {

        “value”: 3.0,

        “units”: “mmol/l”,

        “system”: “http://unitsofmeasure.org”,

        “code”: “mmol/l”

      },

      “high”: {

        “value”: 6.1,

        “units”: “mmol/l”,

        “system”: “http://unitsofmeasure.org”,

        “code”: “mmol/l”

      }

    }

  ]

}

#6–FHIR Resource type: Questionnaire

{

“resourceType”:”QuestionnaireResponse”,

“id”: “UIA101”,

“status”: “completed”,

“authored”: “2021-21-10T00:00:00+01:00”,

“subject”: {

    “reference”: “Patient/example”,

    “type”: “Patient”

    },

“item”: [

    {

        “linkId”: “hasHeight”,

“code”: [

                    {

                      “system”: “http://snomed.info/sct”,

       “code”: “50373000”,

                    }

                ],

        “text”: “Height of Patient in cm”,

        “answer”: [

            {

                “valueDecimal”: 171.0

            }

        ]

    },

    {

        “linkId”: “hasWeightKG”,

“code”: [

                    {

                      “system”: “http://snomed.info/sct”,

        “code”: “64305001”,

                    }

                ],

        “text”: “Initial Weight of Patient in KG”,

        “answer”: [

            {

                “valueDecimal”: 70.7

            }

        ]

    },

    {

        “linkId”: “hasSnus”,

“code”: [

                    {

                      “system”: “http://snomed.info/sct”,

        “code”: “713914004”,

                    }

                ],

        “text”: “Quantity in number”,

        “answer”: [

            {

                “valueDecimal”: “0.0”

            }

        ]

    },

    {

        “linkId”: “hassmoked”,

“code”: [

                    {

                      “system”: “http://snomed.info/sct”,

        “code”: “365981007”,

                    }

                ],

        “text”: “Quantity in number”,

        “answer”: [

            {

                “valueDecimal”: “0.0”

            }

        ]

    },

    {

        “linkId”: “hasAlcohol”,

“code”: [

                    {

                      “system”: “http://snomed.info/sct”,

       “code”: “228281002”,

                    }

                ],

        “text”: “quantity in ml.”,

        “answer”: [

            {

                “valueDecimal”: “50”

            }

        ]

    },

    {

        “linkId”: “hasFastFood”,

“code”: [

                    {

                      “system”: “http://snomed.info/sct”,

       “code”: “230112002”,

                    }

                ],

        “text”: “Confectionery intake in gm.”,

        “answer”: [

            {

                “valueDecimal”: “20”

            }

        ]

    },

    {

        “linkId”: “hasVegetables”,

“code”: [

                    {

                      “system”: “http://snomed.info/sct”,

       “code”: “226448008”,

                    }

                ],

        “text”: “Vegetables in gm.”,

        “answer”: [

            {

                “valueDecimal”: “300”

            }

        ]

    },

    {

        “linkId”: “hasSalad”,

“code”: [

                    {

                      “system”: “http://snomed.info/sct”,

       “code”: “227927005”,

                    }

                ],

        “text”: “Green Salad in gm.”,

        “answer”: [

            {

                “valueDecimal”: “200”

            }

        ]

    },

    {

        “linkId”: “hasFruits”,

“code”: [

                    {

                      “system”: “http://snomed.info/sct”,

        “code”: “72511004”,

                    }

                ],

        “text”: “Fruits in gm.”,

        “answer”: [

            {

                “valueDecimal”: “100”

            }

        ]

    },

    {

        “linkId”: “hasSweetBeverages”,

“code”: [

                    {

                      “system”: “http://snomed.info/sct”,

       “code”: “818989004”,

                    }

                ],

        “text”: “Sweet Beverages in ml.”,

        “answer”: [

            {

                “valueDecimal”: “30.0”

            }

        ]

    },

    {

        “linkId”: “hasActivity”,

“code”: [

                    {

                      “system”: “http://snomed.info/sct”,

       “code”: “68130003”,

                    }

                ],

        “text”: “Activity Duration in hr.”,

        “answer”: [

            {

                “valueDecimal”: “1.5”

            }

        ]

    },

{

        “linkId”: “hasTypeActivity”,

“code”: [

                    {

                      “system”: “http://snomed.info/sct”,

       “code”: “280147009”,

                    }

                ],

        “text”: “Activity Type”,

        “answer”: [

            {

                “valueString”: “Walking”

            }

        ]

    },

    {

        “linkId”: “hasHealthStatus”,

        “text”: “Health Status of Patient”,

        “answer”: [

            {

                “valueString”: “Postcode”

            }

        ]

    },

    {

        “linkId”: “hasHealthStatus”,

“code”: [

                    {

                      “system”: “http://snomed.info/sct”,

       “code”: “263490005”,

                    }

                ],

        “text”: “Status of Partcipant”,

        “answer”: [

            {

                “valueString”: “Healthy”

            }

        ]

    },

    {

        “linkId”: “durationOfSurvey”,

“code”: [

                    {

                      “system”: “http://snomed.info/sct”,

        “code”: “103335007”,

                    }

                ],

        “text”: “Survey Duration”,

        “answer”: [

            {

                “valueInteger”: 0

            }

        ]

    },

    {

        “linkId”: “satisfactionLevel”,

“code”: [

                    {

                      “system”: “http://snomed.info/sct”,

       “code”: “405153007”,

                    }

                ],

        “text”: “Satisfaction Level”,

        “answer”: [

            {

                “valueInteger”: 0

            }

        ]

    },

    {

        “linkId”: “terminationDate”,

        “text”: “Termination Date”,

        “answer”: [

            {

                “valueDateTime”: “2021-07-10T00:00:00+01:00”

            }

        ]

    }

  ]

}.
